# The GrimAge clock corresponds to brain ageing and social determinants of health in people with HIV

**DOI:** 10.1016/j.ebiom.2026.106492

**Published:** 2026-09-24

**Authors:** Kalen J. Petersen, Seungjae Kim, Pete Canfield, June Roman, Sarah A. Cooley, Manjula Bhuma, Adam Anderson, Brittany Nelson, Patricia Reid, Elizabeth Westerhaus, Maurizio Caocci, Tricia H. Burdo, Aristeidis Sotiras, Beau M. Ances

**Affiliations:** a Department of Neurology, Washington University School of Medicine, St. Louis, MO, USA; b Johns Hopkins University, Baltimore, MD, USA; c Rutgers Institution of Translational Medicine and Sciences, Rutgers, The State University of New Jersey, New Brunswick, NJ, USA; d Department of Radiology, University of Pennsylvania, Philadelphia, PA, USA

**Keywords:** HIV, Human immunodeficiency virus, Neuroimaging, Neuropsychiatry, Epigenetic clock

## Abstract

**Background:**

DNA methylation (DNAm)-based epigenetic clocks can measure biological ageing. However, newer epigenetic clocks sensitive to morbidity, mortality, and longitudinal change have not been compared to brain health biomarkers in virally suppressed people with HIV (PWH) and without HIV (PWoH).

**Methods:**

Participants in this case-control study underwent blood draws, brain MRI, and neuropsychological testing. Four DNAm-based epigenetic clocks were calculated: GrimAge (mortality-predictive), PhenoAge (morbidity-sensitive), DunedinPACE (longitudinal), and DNAm telomere length. Clocks were correlated with MRI features and cognitive domains. Neighbourhood socioeconomics, unemployment, early life stress, education, and medical comorbidities were examined as risk factors for epigenetic ageing.

**Findings:**

126 virologically suppressed PWH (age = 53.8 ± 12.2 yr) and 145 PWoH (age = 48.1 ± 16.4) participated. Elevated GrimAge (*p* < 0.001; +1.8 years), PhenoAge (*p* = 0.002; +2.4 years), and DunedinPACE (*p* < 0.001; +5.1% ageing rate) were found for PWH vs. PWoH, controlling for demographic factors. Predicted telomere length was shorter for PWH vs. PWoH (*p* < 0.001). GrimAge correlated with brain age gap (*p* = 0.002; +5.9 years of brain age per decade of GrimAge) across all participants. Epigenetic clocks were linked to poorer cognition in specific domains. Area Deprivation Index was positively associated with GrimAge (*p* = 0.001) in PWH, while education was negatively associated (*p* = 0.002). Cardiovascular risk was associated with increased GrimAge (*p* = 0.008) across all participants.

**Interpretation:**

Biological age alterations persist in PWH despite viral suppression, as revealed by multiple epigenetic clocks. GrimAge and other clocks are linked with brain morphology and cognitive dysfunction. Results suggest that biological ageing is influenced by modifiable social and comorbid risk factors.

**Funding:**

United States National Institutes of Health (National Institute of Mental Health, National Institute on Drug Abuse, National Institute of Nursing Research) and amfAR, The Foundation for AIDS Research.

**Research in context**

**Evidence before this study**

A PubMed search was used to identify all relevant original research studies published since 2013 when the original Horvath clock was published. Results contained both “HIV” and one or more of the following search terms in the title or abstract: “DNA methylation clock”/“DNAm clock” “epigenetic clock,” “GrimAge,” “PhenoAge,” “DunedinPACE,” and “DNAmTL,” i.e., DNA-methylation telomere length. This resulted in an initial 38 hits, which were then manually screened for relevance to the enclosed study. Five studies were systematic reviews, meta-analyses, or commentaries, and were excluded, as were four studies of paediatric populations. Two studies proposing novel DNA methylation clocks were also omitted.

The following themes emerged from the remaining 27 studies: (1) that epigenetic clocks in blood and other tissues are typically increased in people with HIV (PWH), but (2) the extent of age alteration is variable and at least partially mitigated by anti-retroviral therapy (ART) and undetectable viral load, though the extent of this improvement varied. Moreover, (3) immunological markers of HIV disease severity, including CD4 cell counts, viral load, and inflammatory biomarkers are frequently correlated with the degree of ageing, but (4) the relative contribution of HIV disease compared to other risk factors, including comorbid diseases and social determinants of health is unclear. Notably, (5) multiple clinical trials have identified epigenetic age as therapeutically modifiable using senotherapeutics and other anti-ageing or anti-diabetic drugs including semaglutide and metformin. However, (6) the relationship between ageing in the periphery and the central nervous system remains poorly understood, as is the relevance of epigenetic clocks to cognitive impairment.

**Added value of this study**

Here, we utilise neuroimaging and DNA methylation clocks to address this gap using a large sample of PWH with viral suppression and controls without HIV. This study adds to the existing literature in several respects: first, it uses disease-sensitive second- and third-generation epigenetic clocks to examine brain health in PWH. Second, it employs both brain MRI and neuropsychological testing to quantify structural and functional brain health in relation to epigenetics. Third, it examines important social and structural factors including education and socioeconomic status as potential factors related to epigenetic ageing in PWH.

**Implications of all the available evidence**

Taken in literary context, our findings point to epigenetic clocks as not only sensitive biomarkers for observational studies of brain health in PWH, but as potentially modifiable outcomes in intervention studies targeting ageing processes and/or mitigating the impact of comorbidities. They also suggest that different DNAm clocks differ in their associations with cognition, physical brain integrity, and social determinants of health. The examination of HIV-related epigenetic changes in neurological disease is a nascent area, but will likely enable us to identify novel mechanisms of central nervous system damage impacting quality of life for older and mid-life PWH.

## Introduction

Several generations of epigenetic clocks are now available to extract information about biological ageing from DNA methylation (DNAm) data.^1^ These models allow measurement of an individual or population’s biological ageing rate by comparison to a model trained on a large sample of typically ageing people. First-generation epigenetic clocks were trained directly on chronological age. In contrast, the second-generation PhenoAge and GrimAge clocks capture phenotypes related to disease burden and mortality, respectively.^2^^,^^3^

Findings from the Veterans Ageing Cohort Study (VACS) demonstrated that DNAm data perform well at predicting mortality in people with HIV (PWH), especially in genes involved in HIV pathogenesis.^4^ GrimAge specifically is strongly predictive of clinical outcomes and all-cause mortality^5^ and resulted in the highest discrimination of mortality risk in VACS.^6^

The third-generation DunedinPACE clock adds a time dimension by predicting longitudinal change rather than a static ‘snapshot’ of biological age.^7^ Additionally, DNAm data can estimate telomere length, a classical marker of cellular ageing altered by disease processes.^8^ DNAm-based telomere length (DNAmTL) is significantly reduced due to HIV and has strong associations with health outcomes including frailty, cancer, and all-cause mortality in PWH.^9^

These epigenetic clocks (Fig. 1A) have been used to show that biological ageing in multiple tissues and organs is increased in PWH compared to people without HIV (PWoH),^10^ and that such ageing can occur while viral load remains undetectable.^11^ However, epigenetic ageing is lessened in those with well-controlled viral load and can approach normal ageing.^12^^,^^13^

**Fig. 1:**
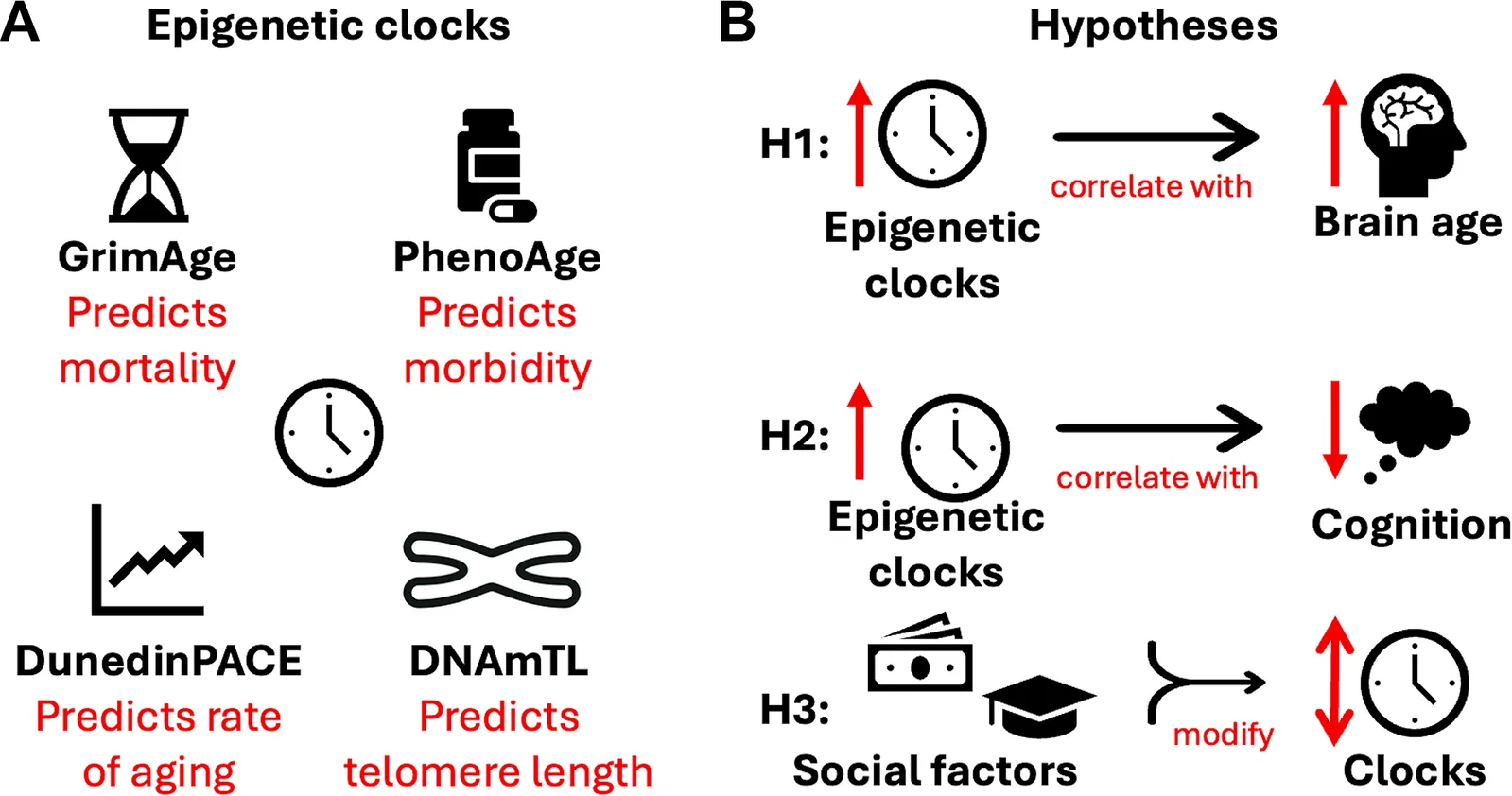
**(A)** DNA methylation (DNAm) clocks and their functional tunings. PhenoAge is sensitive to multi-organ disease burden, GrimAge predicts mortality, DunedinPACE estimates longitudinal ‘pace of ageing’ from a single timepoint, and DNAmTL measures telomere length from DNAm data. **(B)** Hypotheses: [1] Epigenetic clocks correlate with magnetic resonance imaging (MRI)-based brain age gap (BAG); [2] Epigenetic clocks are associated with cognitive dysfunction; and [3] Epigenetic clocks are affected by social determinants of health (socioeconomic status, education) in PWH and PWoH.

Some longitudinal studies have found variation in DNAm response to combination anti-retroviral therapy (cART), with a majority of treatment-naïve PWH showing improved epigenetic age after cART initiation, though a minority had increases in estimated age.^14^ However, a recent study of PWH with sustained viral suppression nonetheless found elevated epigenetic age associated with risk for serious non-AIDS health events, ageing-related events, and mortality.^15^

Though altered DNAm may be partly attributed to HIV, the contributions of comorbid disease^16^ and environmental factors^17^ may also dysregulate epigenetics. Notably, epigenetic ageing has been associated with social determinants of health including protective (e.g., education) and deleterious factors (e.g., lower socioeconomic status).^18^

Epigenetic age in PWH and PWoH has been linked with neurocognitive impairment in multiple functional domains, including working memory, spatial processing, motor coordination, and executive function.^19^ Newer clocks with superior sensitivity to disease may improve our ability to examine HIV effects on the neurobiological processes underlying cognitive impairment and decline.

However, despite the availability of this rich toolkit, only first-generation clocks have been associated with neuroimaging biomarkers in PWH. Several important questions remain unanswered, including whether newer clocks are altered in PWH with undetectable viral load, whether they are related to brain structural and functional ageing, whether due to HIV or to external factors such as socioeconomic conditions and comorbidities.

We seek to address these gaps through a comparison of second- and third-generation epigenetic clocks with brain ageing and cognition. To quantify brain ageing, magnetic resonance imaging (MRI) is used to calculate ‘brain-predicted age’ based on a model previously trained on large neuroimaging datasets. Analogous to epigenetic clocks, this technique compares an individual’s brain structure to a population norm to yield the brain age gap (BAG).

The BAG represents the deviation of an individual’s apparent brain age from the typical trajectory. By combining epigenetic clocks and BAG, we can estimate relationships between biological ageing in the peripheral epigenome and central nervous system, including brain atrophy and cognitive impairment. We then test whether socioeconomic deprivation, unemployment, lower education, early life stress, and medical comorbidities are risk factors for epigenetic ageing.

## Methods

### Participants

De-identified blood from adult PWH and PWoH was taken from studies performed at Washington University in Saint Louis School of Medicine between 2012 and 2024. Participants were recruited through the Washington University Infectious Diseases Clinic or via research fliers. These studies included brain MRI and neuropsychological testing and were performed by the same team, at a single visit, with consistent methodology. HIV serostatus was positively confirmed for PWH using reverse-transcriptase PCR, PWoH were confirmed as seronegative via rapid oral swab testing. Sample sizes were determined by blood sample availability for participants meeting enrolment criteria.

### Ethics

All participants provided informed consent to the original study procedures and the use of data and tissues for subsequent studies per the Washington University IRB (approval numbers 202603137, 202409207, 202202167, 202107149, 201805047, 201508002, 201212020, 201204041). This study conforms to the Declaration of Helsinki on human research ethics.

Exclusion criteria for both PWH and PWoH were diagnosis of non-HIV-related neuropsychiatric disorders, history of traumatic brain injury with unconsciousness, opportunistic brain infection, current substance use disorder interfering with participation, and untreated major depression. People with mild-to-moderate depression or mood disorders, and those with light-to-moderate substance use were included to increase external validity, given the prevalence of these factors.

Lifetime substance use was quantified using the Kreek–McHugh–Schluger–Kellogg (KMSK) scale. Exposure to childhood or adolescent stressors was quantified using the Early Life Stress Questionnaire in a subset of 43 participants. Employment status was self-reported. Cognitive testing consisted of a battery of 12 tests in five domains (Table 1). Domain *z*-scores were obtained as the mean of test-specific *z*-scores after normalisation, following the published instructions for each constituent test.

**Table 1:** Neurocognitive battery components.

Domain	Tests
Psychomotor	Trails A, Digit Symbol, grooved pegboard, Symbol Search
Executive	Colour word interference (trial 3), verb fluency, Trails B, letter number sequencing
Language	Letter fluency (FAS), category fluency (animals)
Learning	Hopkins verbal learning test, brief visuospatial memory task
Memory/recall	Hopkins verbal learning test, brief visuospatial memory task

PWH were required to be on stable cART with plasma viral suppression to <200 copies/mL for at least three months. CD4 and CD8 T-cell counts and their ratio were obtained using Quest Diagnostics’ Lymphocyte Subset Panel 4 (test code 7924) in Wood Dale, Illinois, a CLIA-certified laboratory. Hepatitis C status and nadir CD4 levels were obtained from clinical records or self-report. Framingham 10-year cardiovascular risk was calculated from age, sex, cholesterol (total and HDL), blood pressure, smoking, and diabetes status.

### DNA methylation clocks

EDTA-anticoagulated blood, collected at the time of neuroimaging, was used to isolate peripheral blood mononuclear cells (PBMCs) using standard Ficoll separation. DNA from PBMCs was extracted using a NucleoSpin mini-kit (Macherey-Nagel, Düren Germany), quantified using a PicoGreen kit (Thermo Fisher, Waltham MA), and normalised to 25 ng/μL. Methylation levels were quantified using MethylationEPICv2 microarrays (Illumina, Inc., San Diego CA). Quantile normalisation was used to correct methylation intensity differences using the *minfi* R package (v1.56.0). Probe annotation was based on the manifest EPIC-8v2-0_A2 with genomic annotation version 20.a1.hg38.

Poor-quality probes were removed prior to analysis by calculation of detection *p*-values using *minfi* tools. Probes with *p*-values > 0.01 were considered undetectable. Any missing probes required for DNA methylation clocks were imputed using the *methylclock* package (v.1.16.0). In addition to per-probe exclusion, bias was reduced through normalisation and correction for background intensity, dye bias, probe-type bias, and batch effects.

DNAm QC thresholds for exclusion were: (1) mean detection *p*-values > 0.05 from *minfi*’s detectionP() function with a control probe-based background distribution using a one-sided upper-tail probability, (2) DNAm-derived chromosomal mismatch with recorded biological sex, (3) failure of control probes for hybridisation, staining, or bisulfite conversion, and (4) low mean probe intensity (methylated or unmethylated). One participant was excluded under category 4. Principal component analysis was used to ensure that no technical or biological outliers remained after QC exclusions.

The principal component-based version of three clocks, GrimAge, PhenoAge, and DNAmTL^20^ were calculated using publicly available R code (v4.4.1) [github.com/MorganLevineLab/PC-Clocks] (v0.1.0). GrimAge is sensitive to mortality; PhenoAge captures multi-system disease morbidity; and DNAmTL targets chromosomal ageing. DunedinPACE was calculated using a separate R package [github.com/danbelsky/DunedinPACE], and is the only clock designed to predict future biological ageing from DNAm at one timepoint. White blood cell proportions were estimated based on reference datasets using the *FlowSorted.Blood.EPIC* R package (v2.14.0).^21^

### Risk factors

Socioeconomic deprivation, early life stress, and unemployment were hypothesised to be risk factors for epigenetic ageing, while education was hypothesised to be protective. Length of formal education was quantified; the Wide Range Achievement Test 3rd edition (WRAT-III) reading subscale was used to confirm that differences in education corresponded to actual differences in attainment.

Neighbourhood socioeconomics were described using the 2019 Area Deprivation Index (ADI) which combines census-tract unemployment, income, housing, and education data into a composite percentile index, in which 100 represents the highest deprivation level.^22^ Unemployment was examined as a discrete predictor. Several common medical comorbidities were tested for association with epigenetic ageing: hepatitis C co-infection, Framingham cardiovascular risk score, and depressive symptoms (Beck Depression Inventory-II).

### Neuroimaging

Brain images for PWH and PWoH were acquired using a 3-T Siemens PRISMA scanner. Brain volume and cortical thickness were obtained using a T_1_-weighted magnetisation prepared rapid gradient echo sequence (repetition time/echo time = 2400/3.2 ms, spatial resolution = 1 × 1 × 1 mm) as described previously.^23^ We utilised minimal processing: non-brain tissue was removed using *Robex* v1.2 [https://www.nitrc.org/projects/robex/], then the brain was linearly registered to the Montreal Neurological Institute 1-mm template using the FMRIB Software Library’s registration tool (FSL v6.0.7.18).

MRI quality control consisted of visual examination for motion artifacts, such as ringing, blurring, or ghosting, susceptibility artifacts (signal dropout or distortion), aliasing, and inhomogeneities. Scans with major artifacts of these kinds were excluded. Registration and brain segmentations were evaluated visually and quantitatively prior to FreeSurfer processing, and outliers or clear cases of misalignment were re-run if possible or excluded if not.

### Brain-predicted age

Processed scans were used to derive brain-predicted age using DeepBrainNet, a convolutional neural network trained on a large MRI cohort across numerous sites^24^ (https://github.com/vishnubashyam/DeepBrainNet). BAG was defined as the difference between a participant’s predicted and chronological age, such that a positive BAG indicates features associated with faster-than-expected ageing, while a negative BAG is interpreted as brain age ‘deceleration’.^25^ To correct for chronological age bias, we included true age as a covariate in all analytical modelling of BAG and epigenetic age.^26^

FreeSurfer corrections were performed by trained and certified technicians blinded to HIV serostatus. Manual adjustments were performed to mitigate the following errors by adjusting tissue boundaries: cerebellum inclusions (i.e., non-brain regions as cerebellum) or exclusions (cerebellum as non-brain), subcortical labelling errors, lateral ventricle inclusions/exclusions, errors in pial surface reconstruction, grey matter inclusions/exclusions, sulcal errors (CSF enclosed by sulci) and white matter segmentation errors. FreeSurfer was re-run up to three times after each round of manual corrections.

### Brain regions

BAG is a whole-brain measure. In contrast, the volume and thickness of individual brain parcels were determined with FreeSurfer (v.7.3) with manual correction by trained technicians. FreeSurfer maps provide volumes across anatomical subdivisions of the cortex and subcortical structures including the basal ganglia, thalamus, and hippocampus. These maps give anatomical context for regionally nonspecific BAG. Volume and thickness estimates were not used in hypothesis testing; however, *t*-scores for the association between volume or thickness and epigenetic clocks are provided as hypothesis-generating data to guide future studies.

### Hypotheses

The following hypotheses were tested (Fig. 1B): [1] epigenetic ageing correlates with faster brain ageing (BAG); [2] epigenetic ageing is associated with impairment in specific cognitive domains (Table 1); and [3] non-HIV social determinants of health (education, socioeconomic deprivation, employment status, and early life stress) and medical comorbidities (hepatitis C, cardiovascular risk, depression) are associated with epigenetic ageing in both PWH and PWoH.

### Statistics

Generalised linear regression models included the following covariates: chronological age, sex, self-identified race, estimated blood cell proportions including CD4 lymphocytes, and microarray plate number (to mitigate batch effects). Associations were significant at an *a priori* threshold of *p* = 0.05. As hypothesis [2] considered five cognitive domains vs. four epigenetic clocks, Benjamini-Hochberg false-discovery rate (FDR) correction was applied across 20 tests. All effect estimates are reported adjusted for covariates unless otherwise stated. Quantitative variables were treated as continuous predictors and were not binned or binarized.

To determine whether the HIV effects on cognition are mediated by epigenetic ageing, we performed mediation analysis for any clock-domain pair with significant unadjusted correlation, testing whether the clock in question carries the indirect statistical effect of HIV serostatus on cognitive function using the *mediation* R package with 1000 bootstraps. In a given mediation model, HIV serostatus was the independent variable, the cognitive domain *z*-score was the outcome variable, and the selected clock was the mediator. Residual normality in the fitted models was confirmed using Shapiro–Wilk tests and visual examination of Q–Q plots.

### Role of funders

Funders had no role in study design, data collection, analysis, interpretation, writing, or decision to publish.

## Results

### Participant demographics

Data from 126 PWH (age = 53.8 ± 12.2 yr; sex = 28% female) and 145 PWoH (48.1 ± 16.4; sex = 46% female) were included. One participant was excluded for poor DNAm data quality. PWH and PWoH differed in chronological age (*p* = 0.001; Student’s t-test), sex (*p* = 0.003; chi-square), and self-identified race (*p* = 0.001; chi-square). For PWH, viral load was 22.6 ± 11.0 copies/mL, and CD4 T cell count was 693 ± 304 cells/μL. The mean time since HIV diagnosis was 18.9 years and the mean duration of cART was 16.1 years (Table 2).

**Table 2:** Participants.

	PWH	PWoH	*p*-value	Test
*N* participants	126	145		
Demographics				
Age (years ± SD)	53.8 ± 12.2	48.1 ± 16.4	0.001	Student’s t-test
Sex			0.003	Chi-square
Male (%)	91 (72)	78 (54)		
Female (%)	35 (28)	67 (46)		
Race			0.001	Chi-square
Black/Afr. Amer. (%)	68 (54)	51 (35)		
White (%)	56 (44)	82 (57)		
Other (%)	2 (2)	12 (8)		
Education (years ± SD)	13.9 ± 2.7	15.4 ± 2.5	<0.001	Student’s t-test
WRAT-III reading score	46.2 ± 8.2	49.9 ± 5.8	<0.001	Student’s t-test
Unemployed or disabled (%)	37 (29)	25 (17)	0.02	Chi-square
Area deprivation index (percentile)	69.4 ± 27.1	57.6 ± 28.3	0.002	Student’s t-test
Early life stress questionnaire^a^	4.4 ± 2.5	1.4 ± 1.2	0.002	Student’s t-test
HIV clinical variables				
Time since diagnosis (years ± SD)	18.9 ± 10.4			
Duration of cART^b^ (years ± SD)	16.1 ± 9.5			
Duration of current HIV regimen^c^ (years ± SD)	4.4 ± 4.2			
HIV viral load (copies/mL ± SD)	22.6 ± 11.0			
CD4 T-cells (cells/μL ± SD)	693 ± 304			
Nadir CD4 T-cells (cells/μL ± SD)	232 ± 201			
CD4/CD8 ratio	1.0 ± 0.5			
Other clinical variables				
Framingham risk score	15.0 ± 10.4	9.7 ± 8.3	<0.001	Student’s t-test
Hepatitis C history (%)	10 (8%)	2 (1%)	0.02	Chi-square
Substance use history				
Cannabis (KMSK ± SD)	4.7 ± 5.0	3.0 ± 4.0	0.008	Student’s t-test
Alcohol (KMSK ± SD)	6.2 ± 4.2	5.9 ± 4.0	0.53	Student’s t-test
Tobacco (KMSK ± SD)	5.4 ± 5.2	2.7 ± 4.3	<0.001	Student’s t-test
Cocaine (KMSK ± SD)	2.5 ± 4.4	0.9 ± 3.2	<0.001	Student’s t-test

a Data available for 43/271 (16%) of participants.

b Data available for 105/126 (83%) of PWH.

c Data available for 94/126 (75%) of PWH.

### Epigenetic clocks

As expected, all four epigenetic clocks correlated with chronological age, though fit and intercept varied (Fig. 2A). DunedinPACE rate of change correlated positively with chronological age (R = 0.41) indicating that epigenetic ageing pace increases with age in both PWH and PWoH.

**Fig. 2:**
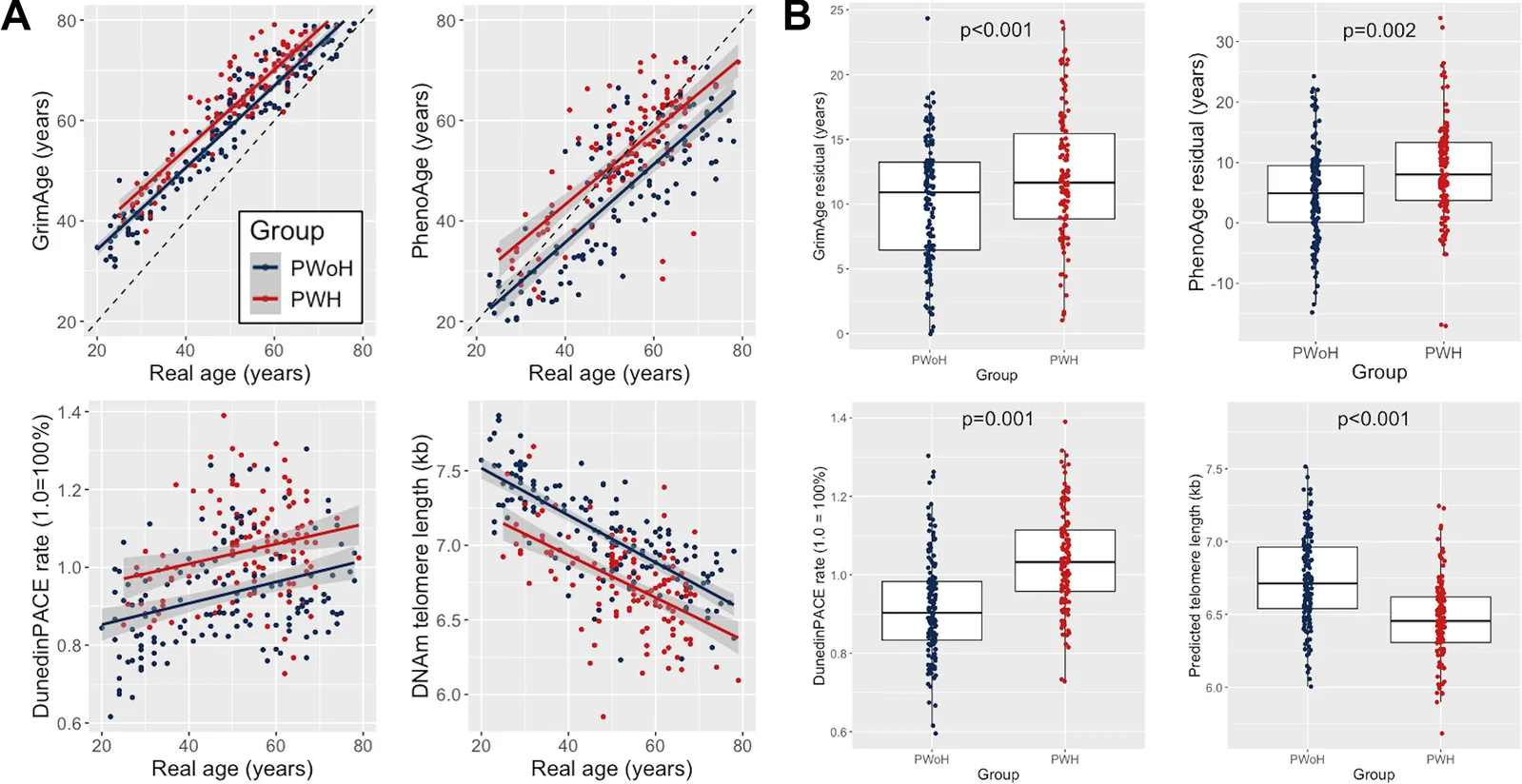
**(A)** Chronological age (years of life) is shown on the *x* axis and Epigenetic clocks are shown on *y*. PhenoAge and GrimAge were highly correlated with chronological age, and DNAmTL was negatively correlated, as expected. DunedinPACE represents ageing rate as a percentage, where 1.0 equals 100% of typical biological ageing. The positive association between age and DunedinPACE indicates that the rate of ageing gradually increases, suggesting acceleration. **(B)** Boxplots representing the distributions of epigenetic age residuals by HIV serostatus for PhenoAge, GrimAge, DunedinPACE (rate of change, where 1.0 represents typical ageing), and DNAm-based telomere length in kilobases (kb). All four clocks were significantly altered in PWH (n = 126) compared to PWoH (n = 145). All *p*-values are from Wald t-tests for coefficients in generalised linear regression models.

We tested whether epigenetic clocks are altered in PWH with viral suppression, consistent with published studies. All analyses were adjusted for chronological age, sex, race, microarray batch, and the proportion of six cell types (CD4 and CD8 T-cells, natural killer cells, B cells, monocytes, neutrophils), to ensure that epigenetic clocks were not biased by differences in cell type. As expected, CD4 T-cells were lower in PWH (*p* < 0.001; Student’s t-test) and CD8 T-cells were more numerous (*p* < 0.001; Student’s t-test).

GrimAge (*p* < 0.001; Wald t-test; +1.8 years), PhenoAge (*p* = 0.002; Wald t-test; +2.4 years), and DunedinPACE (*p* < 0.001; Wald t-test; +5.1% rate) were elevated in PWH compared to PWoH after accounting for covariates, while DNAmTL was shorter for PWH despite viral suppression (*p* < 0.001; Wald t-test) (Fig. 2B). We conclude that PWH with well-controlled HIV experience an approximately 2-year increase in ageing, a 5% increase in the rate of ageing, and significant telomeric shortening across white blood cell types. The HIV effect on GrimAge persists, and slightly increases from +1.8 years to +2.0 years, when controlling for both education and cardiovascular risk (10-year Framingham score).

### Hypothesis 1: epigenetic clocks are associated with brain ageing

We tested the hypothesis that epigenetic age acceleration is correlated with increased MRI-based brain age gap (BAG) across both PWH and PWoH, and separately in each group.

Overall, increased BAG was associated with greater GrimAge (+5.9 years BAG/decade GrimAge; *p* = 0.002; Wald t-test), indicating that older-appearing brain phenotypes are correlated with mortality-predictive epigenetic patterns. BAG was significantly associated with GrimAge in PWoH alone (+7.1 years/decade; Wald t-test; *p* = 0.01). While the effect size was similar in PWH (+4.7 years/decade), this did not reach statistical significance (*p* = 0.11; Wald t-test).

BAG was also positively associated with DunedinPACE across all participants (+1.0 years BAG/10% DunedinPACE ageing; *p* = 0.037; Wald t-test). DunedinPACE did not reach significance for PWH or PWoH alone. PhenoAge and DNAmTL were not associated with BAG in PWH, PWoH, or the combined sample.

Given the association between GrimAge and BAG, we measured correlations between GrimAge and the volume and thickness of brain regions across all participants. Increased GrimAge was associated with reduced volume in 81 out of 93 regions, with maximum reduction in the medial frontal lobe. Fig. 3A depicts this pattern, with darker red indicating a more negative association. When stratifying by HIV serostatus, we found similar global effects, with GrimAge being associated with reduced volumes for both PWH and PWoH (Supplementary Fig. S1). The former group trended toward frontal lobe reductions, and the latter toward more posterior and subcortical effects.

**Fig. 3:**
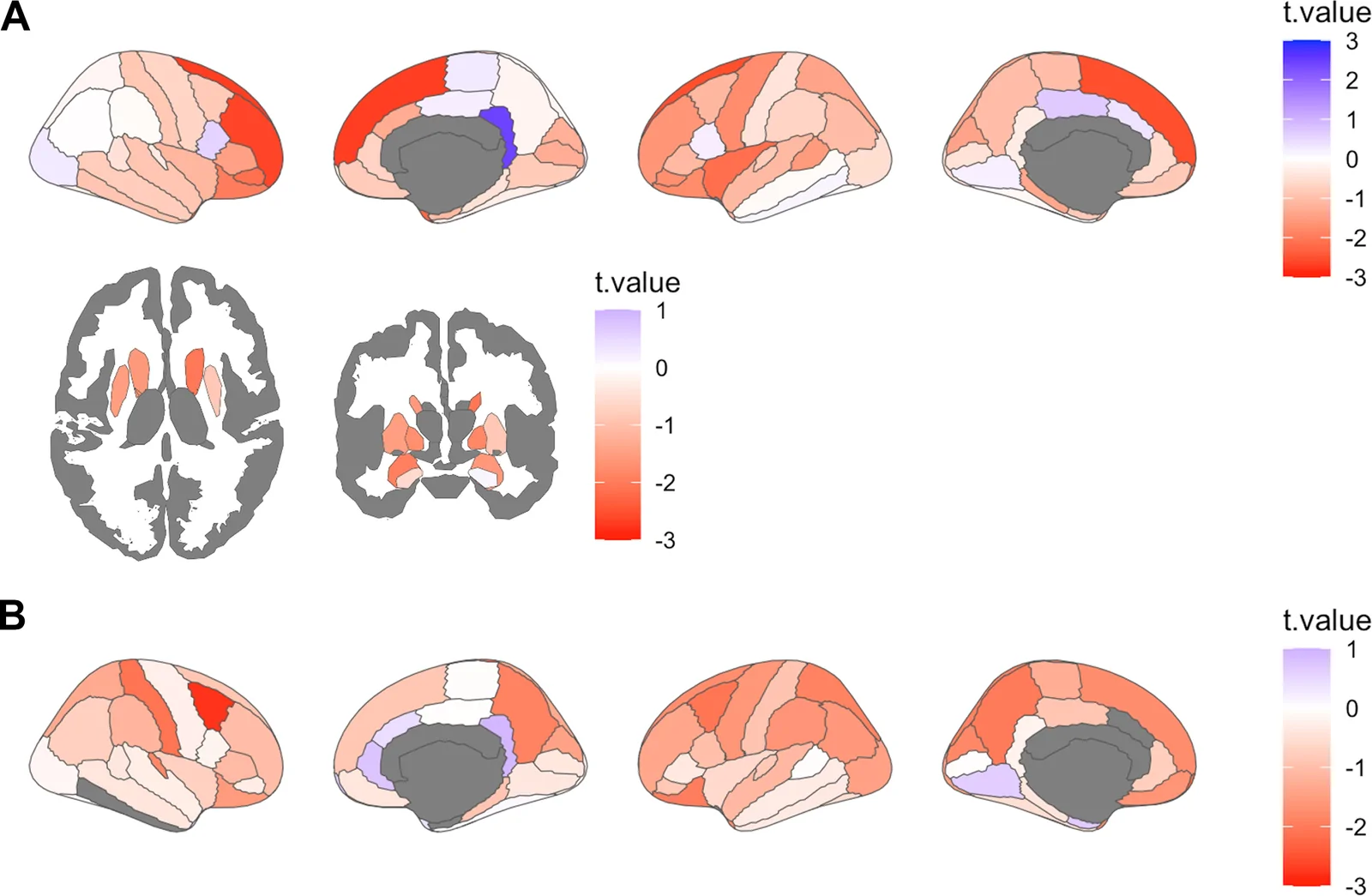
GrimAge was correlated with changes in brain volume and thickness across all participants (n = 126 PWH; n = 145 PWoH). **(A)** GrimAge vs. Regional Volume. Association of GrimAge with regional parcellations of the cerebral cortex, basal ganglia, and limbic system are depicted with a red-blue heatmap. A darker red colour indicates a more negative association between GrimAge and the volume of the individual brain region across all participants, represented as a t-score (t.value). **(B)** GrimAge vs. Cortical Thickness. Association of GrimAge with regional cortical thickness. Negative correlations between cortical thickness and GrimAge in a given parcel are depicted in red, positive associations in blue. Parcels not examined are greyed.

We also observed reduced grey matter thickness with increased GrimAge in 59 of 70 cortical regions, with the most negative association in the frontal and parietal cortex (Fig. 3B; darker red indicates a stronger negative correlation between cortical thickness and GrimAge). Cortical thickness was more negatively associated with GrimAge for PWoH than PWH (Supplementary Fig. S2) suggesting that regional volume is more sensitive to GrimAge than grey matter thickness in PWH. These correlations were intended to be hypothesis-generating and we did not perform hypothesis testing on regional measures.

### Hypothesis 2: epigenetic clocks negatively correlate with cognition

We tested the hypothesis that epigenetic age acceleration is associated with cognitive impairment in discrete domains (Table 1, Supplementary Fig. S3). We compared results from four clocks with five domains (executive function, learning, recall, psychomotor slowing, and language), demographically normalised.

GrimAge was correlated with worse executive function across all participants, though only at a trend level after FDR correction (*p* = 0.01; *p*_adj_ = 0.07; Wald t-test) (Fig. 4A). GrimAge was associated with executive function in PWoH (*p* = 0.02; Wald t-test) but not PWH (*p* = 0.13; Wald t-test), though effect sizes were similar at approximately −0.3 cognitive z-units/decade GrimAge. Mediation analysis showed that GrimAge carried the indirect statistical effect of HIV on executive function (*p* = 0.01; nonparametric bootstrap), though given the simultaneous acquisition of these data, a causal effect cannot be inferred. GrimAge was also nominally associated with worse language function in all participants (*p* = 0.02; Wald t-test) and PWoH (*p* = 0.02; Wald t-test) but *p*-values did not survive FDR correction.

**Fig. 4:**
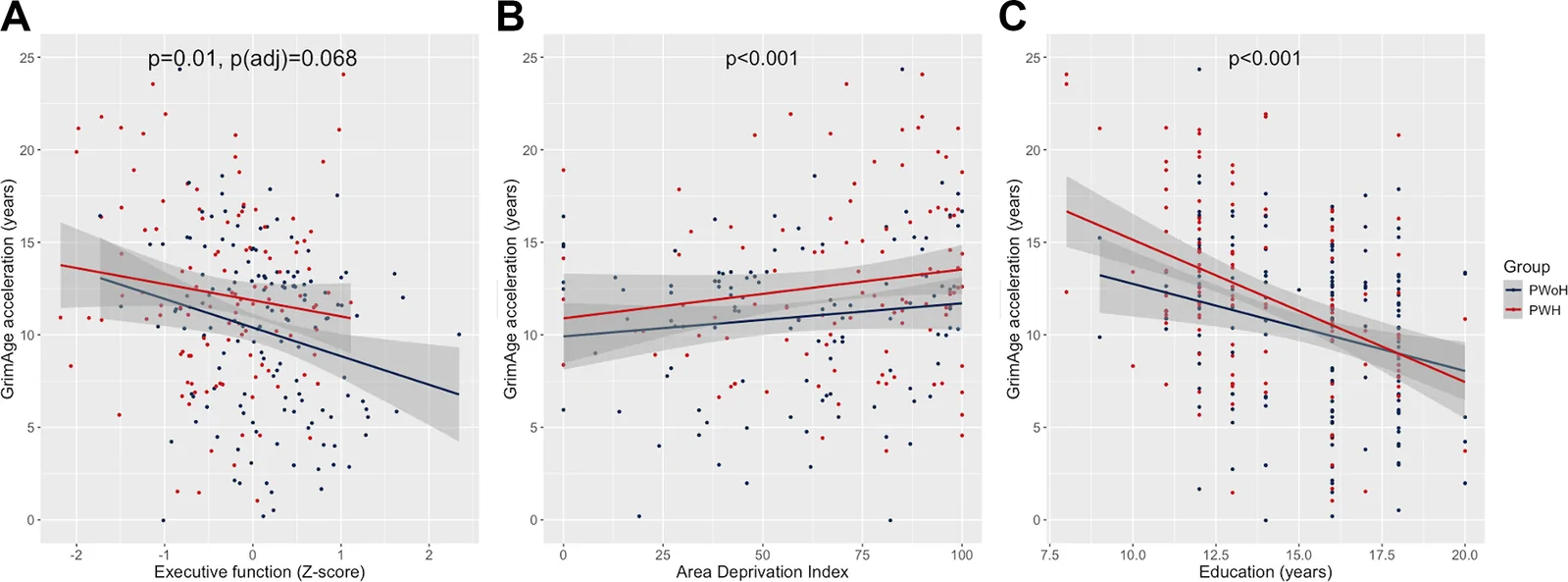
**(A)** Increases in GrimAge were associated with reduced executive function across all participants (n = 126 PWH; n = 145 PWoH). Models were adjusted for demographics and estimated blood cell composition, though this was not significant after correction for multiple comparisons across clocks and cognitive domains (*p* = 0.01; *p*_*adj*_ = 0.07; Wald t-test). **(B)** Area Deprivation Index (ADI) was significantly associated with greater GrimAge (*p* < 0.001; Wald t-test), while **(C)** education was associated with reduced GrimAge (*p* < 0.001; Wald t-test).

Higher PhenoAge was associated with reduced executive function across participants (*p* = 0.01; Wald t-test) but this also fell short of significance after FDR correction (*p*_adj_ = 0.07). This effect was similar in PWH (*p* = 0.02; Wald t-test) but not in PWoH (*p* = 0.14; Wald t-test), suggesting an HIV-specific association.

DunedinPACE was nominally associated with language across participants, but this did not survive FDR correction (*p* = 0.01, *p*_*adj*_ = 0.07; Wald t-test), nor did the effect for PWH or PWoH alone. DNAmTL was the only epigenetic clock with significant cognitive association after FDR correction, namely language function (*p* < 0.001; *p*_adj_ < 0.001; Wald t-test), and this negative association was driven by PWoH (*p* = 0.005; Wald t-test).

### Hypothesis 3: social determinants of health and comorbidities are associated with epigenetic ageing

Socioeconomic deprivation, unemployment, early life stress, unemployment, education, and common clinical comorbidities were examined as risk factors for DNAm ageing.

We found that increased ADI (greater socioeconomic deprivation) was correlated with GrimAge across all participants (*p* < 0.001; Wald t-test) such that every decile increase in ADI corresponded to +0.22 years of age acceleration (Fig. 4B). Stratified by HIV serostatus, this effect was significant for PWH (+0.34 years/decile ADI; *p* = 0.001; Wald t-test) but not PWoH (*p* = 0.09; Wald t-test).

Conversely, each year of additional education was associated with 0.2 years of reduced GrimAge overall (*p* < 0.001; Wald t-test) (Fig. 4C). Stratified by HIV serostatus, this effect was likewise specific to PWH with an effect of 0.3 years of reduced GrimAge per year of education (*p* = 0.002; Wald t-test) and not significant in PWoH (*p* = 0.14; Wald t-test). Unemployment and early life stress were not associated with GrimAge, though early life stress was only available for a subset.

Among the medical comorbidities, higher 10-year Framingham cardiovascular risk was associated with greater GrimAge overall (+0.77 years GrimAge/10-point increase in Framingham risk; *p* = 0.008; Wald t-test). This effect was not significant in PWH alone, though the effect size was similar (+0.55 years/10-point increase; *p* = 0.20; Wald t-test), but was significant for PWoH (+1.2 years/10-point increase; *p* = 0.005; Wald t-test). Hepatitis C (*p* = 0.56; Wald t-test) and depressive symptoms (*p* = 0.96; Wald t-test) were not associated with GrimAge for either group.

## Discussion

The objective of this study was to measure relationships between biological brain ageing and epigenome in virally suppressed PWH using both next-generation epigenetic clocks sensitive to morbidity, mortality, and longitudinal change, and high-resolution brain MRI. Consistent with the literature, multiple DNAm-based epigenetic clocks including GrimAge, PhenoAge, and DunedinPACE were elevated in PWH despite viral suppression, while DNAm-based telomere length was shortened. Several social determinants of health (lower education, greater ADI) were linked with increased epigenetic age in PWH, while cardiovascular risk was associated with increased epigenetic age in PWoH.

We identified a positive correlation between the GrimAge mortality clock and BAG, a well-validated MRI metric derived from machine learning. GrimAge was nominally associated with executive dysfunction, a hallmark of HIV-associated cognitive disorders, and mediated the adverse effect of HIV on this domain. This indirect effect is consistent with, but does not prove, a causal role for brain ageing in bridging epigenetic changes and cognitive impairment. Other clock-domain relationships were also observed, including DNAmTL with language function. We found that two non-HIV factors oppositely influence age acceleration: neighbourhood-level socioeconomic deprivation was associated with higher GrimAge, and education with lower GrimAge.

Given the presumptive biological sequence in which HIV infection is upstream of immune system epigenetic changes and systemic chronic inflammation, which in turn is upstream of neural injury, accelerated brain ageing, and cognitive symptoms, it is necessary to establish stronger causal mediation evidence through longitudinal follow-up. We are presently acquiring inflammatory biomarkers including several years of follow-up proteomics in a subset of the participants from the present study and plan to monitor their cognitive and neurological health at subsequent time points.

The finding that multiple epigenetic clocks are increased in virally controlled PWH is consistent with previous literature,^12^^,^^27^ though we extend this finding into more recent clocks. Undetectable viral load may be incompletely neuroprotective due to HIV reservoirs and low-level viral replication in the central nervous system. Chronic inflammation may also damage neurons and glia, leading to subtle progressive neurodegeneration and ventricular enlargement.^28^ This explanation is supported by the finding that epigenetic age in PWH is greater when pro-inflammatory genetic variations lead to methylation differences.^29^ It is also supported by evidence from a non-HIV study of the Lothian Birth Cohort linking altered DNAm in inflammatory pathways (C-reactive protein) with cognitive function, effects mediated by brain ageing.^30^

The network of connections between the immune system, the epigenome, and mental disorders including cognitive impairment is complex and may involve other systems such as the gut-brain axis. Viral disruptions of these networks are known to produce heightened inflammatory responses including hepatitis B and the Epstein–Barr virus, which in turn influence neuropsychiatric outcomes.^31^

The role of DNAm in the pathways connecting systemic inflammation and brain health is also evident in a recent study^32^ using both proteomics and epigenetic profiling to link ageing-related brain atrophy with DNAm surrogate of C-reactive protein (CRP) and growth differentiation factor 15 (GDF15). This work showed that several brain regions have lower volumes and faster atrophy when epigenetic markers of inflammation are elevated, and that cognitive impairment and dementia risk are both increased in such cases.

While ongoing pathology contributes to ageing, our findings also raise the idea of legacy effects on the brain and epigenome. This possibility is supported by evidence that cognitive impairment typically follows a stable trajectory after cART initiation,^33^ and that HIV-related differences in brain volumes and cortical thickness are likewise stable.^34^ In the case of this study, most participants had initiated cART decades before, though we lacked data on any periods of treatment discontinuity or viral escape. Extended exposure to earlier, more neurotoxic ART, single-drug therapies, and delays in treatment may also influence the current findings.

The epigenetic clocks examined here are tuned to distinct aspects of biological ageing. PhenoAge was trained on chronic disease-associated blood biomarkers, converting these values into a composite ‘phenotypic age’.^2^ In contrast, GrimAge was trained on mortality curves to estimate the likelihood of death from any cause.^3^ DunedinPACE is unique in its prediction of longitudinal progression from the baseline epigenome, and thus is best suited for prognostic individual ‘pace of ageing’.^7^ DNAmTL, which estimates telomere length directly from DNAm data,^8^ captures cellular ageing through the well-understood chromosomal shortening process.

The convergence of these disparate clocks toward increases in PWH with viral suppression suggests that multiple systems are involved in HIV-related biological ageing. However, the surprising finding that the non-GrimAge clocks (PhenoAge, DunedinPACE and DNAmTL) did not correlate as strongly with BAG may indicate that a distinct set of genomic methylation sites are altered with central nervous system injury, either in brain or in blood. Because the brain is an immune-privileged compartment with differences in host epigenetics and viral evolution,^35^ follow-up studies using post-mortem brain samples may be needed to examine CNS epigenetics and test whether DNAm changes in the brain are related to heterogeneous cognitive impairment.^36^

This study is distinguished by the linkage of next-generation epigenetic clocks with BAG, a machine-learning measure of brain health. BAG was derived from DeepBrainNet, a robust MRI model of lifespan brain changes which was built using a large multisite cohort with over 10,000 scans.^24^ It is thus robust to variations in brain structure across individuals and populations. It is sensitive to ageing in other diseases, including neurological disorders such as Alzheimer’s disease^37^ and has previously been examined in neurological HIV.^23^^,^^25^ However, DeepBrainNet has to our knowledge not been combined with newer epigenetic clocks in the context of HIV.

Although all clocks showed significant worsening in people with well-controlled HIV, controlling for demographics, white blood cell counts, and factors such as education and heart disease risk (in the case of GrimAge), it is notable that they differed in their correlations with both BAG and cognition. For example, PhenoAge but not the other clocks was significantly associated with worse executive function specifically in PWH. Likewise, DunedinPACE and GrimAge, but not PhenoAge or DNAmTL, correlated with BAG in this study. This divergence in results likely points to differences in the design and purpose of each clock.

GrimAge in particular was designed to capture aspects of abnormal ageing including inflammation via a two-step training process in which DNAm models of multiple plasma biomarkers were developed, including GDF15, then combined into a single mortality model. Thus, GrimAge is more sensitive to aberrant blood protein levels including cytokines promoting neuroinflammation.^3^ GrimAge has previously been identified as having stronger correlations with brain ageing than other epigenetic clocks.^38^

Likewise, DunedinPACE has demonstrated strong repeatable associations with worse brain health in midlife and older age, consistent with this clock’s tuning to predicted future DNAm ageing, suggestive of ongoing injury.^39^ This stands in contrast to DNAmTL, which is specifically designed to measure telomere length and not inflammaging, and to PhenoAge, which was trained to estimate comorbidity counts, physical functioning, and survival from blood biomarkers.

The correlation between the BAG and the mortality-sensitive GrimAge suggests that neurological injury is a mechanism for increased mortality and chronic disease risk in older PWH. It also suggests that HIV may drive cognitive impairment in part through age-related DNAm changes. However, it is important to note that this correlative analysis does not establish a directional connection between epigenetics and brain ageing, or test whether both are driven by a third factor such as inflammation. However, we identified GrimAge as a significant mediator of reduced executive dysfunction in PWH, suggesting some degree of causal involvement. Epigenetic clocks may thus be informative biomarkers for clinical trials of neuroprotective drugs and senotherapeutics in PWH.

The data showing that GrimAge is associated with marginally worse executive function and language, while DunedinPACE and DNAmTL were correlated with language, suggest that the epigenetic ageing is relevant to several higher cognitive faculties. Given that the correlation between executive function and GrimAge fell just short of statistical significance after multiple comparisons correction, confirmation in other cohorts is recommended to validate this finding. Executive dysfunction is a key feature of HIV-associated deficits and is sensitive to frontal lobe pathology. Interestingly, higher GrimAge was correlated with reduced brain volume and cortical thickness in areas such as the medial prefrontal cortex. These regions are crucial for decision-making, reward valuation, and top-down cognitive control.^40^

Not all epigenetic differences between PWH and PWoH should be attributed directly to the primary effects of HIV. It is crucial to consider the impact of societal factors linked with HIV-positive status.^41^ In this study we tested the hypothesis that DNAm ageing in PWH and PWoH would be affected by non-AIDS risk factors; specifically the Area Deprivation Index, unemployment, early life stress, and education. We found that neighbourhood-level impoverishment was associated with increased GrimAge, portending increased biological ageing and mortality risk, while education corresponded to reduced GrimAge. Similarly, we found that heart disease risk was linked with greater GrimAge, suggesting that non-AIDS factors that remain more common in PWH may explain disparities in ageing.

This study thus supports examining social and structural determinants of health alongside comorbidities of HIV to understand biological ageing. It also suggests that policy-based interventions to mitigate disparities may be effective in reducing health gaps between PWH and PWoH. Other lifestyle factors related to social network size and social engagement or isolation should be considered to understand biological and brain ageing; though beyond the scope of the present study, these have been reviewed elsewhere.^42^

Several limitations must be considered when interpreting this study. First, epigenetic ageing could be attributed in part to cohort effects: older PWH were likely exposed to longer periods of uncontrolled viraemia, more toxic forms of cART, more numerous or severe comorbidities, and greater legacy effects. Latent differences in exposures by cohort must be considered as potential alternatives for the apparent age-increasing effects of HIV in this cross-sectional analysis.

Second, while epigenetic clocks predict health outcomes and disease progression, all these clocks are nonetheless correlative. In other words, it is unclear whether the epigenetic changes selected by these models are ‘drivers’ of ageing, benign ‘passengers,’ or even positive adaptational responses to disease. Each clock likely includes some combination of the foregoing. Future work will examine the relationships between neurological HIV, brain ageing, and more recent epigenetic clocks such as CauseAge and AdaptAge, which are enriched for causal or adaptive epigenetic changes.

Third, this study was limited by its cross-sectional design, which does not allow assessment of stability or reproducibility of clocks. However, extant literature suggests that epigenetic ageing, while highly reproducible, is also responsive to relatively short-term lifestyle changes such as cART initiation.^12^^,^^13^

Fourth, while we were able to examine cognitive status in multiple domains using an extensive neuropsychological battery, there is a growing research focus on participants’ subjective cognitive complaints in areas like memory loss or activities of daily living.^43^ The present study was not designed to quantify these important subjective dimensions of cognitive health.

Finally, this study was limited to epigenetics from blood rather than from brain. HIV has been previously linked with increased epigenetic age in nervous system tissue, and such effects are linked to premortem neurocognitive disorders.^44^ Our epigenetic measurements are less directly related to brain health, but correlations between epigenetic clocks and cognitive function or brain MRI features suggest that these peripheral measures may still be useful for neurological studies. As blood samples are more readily available in large clinical and research cohorts, their use may increase the generalisability of our findings to populations for whom brain health measures such as MRI are unavailable.

The implications of the present work and other studies of epigenetic ageing for clinical care of PWH remain somewhat uncertain.^45^ Genome-wide DNA methylation profiling remains too expensive for routine clinical use, though the advance of higher-throughput methods with robotic sample handling is likely to reduce future costs. However, DNAm profiling is also more accurate and reliable than older measures like telomere length measurement, and yields more insights into the regulation of specific genes and pathways.

One potential area of application is risk prediction for hard clinical endpoints such as coronary events, frailty, cancer, and all-cause mortality in PWH, as has been shown in the VACS cohort.^9^ The minimally invasive approach of using blood rather than solid tissue biopsy makes more routine clinical use more plausible, though potentially less informative for other organ systems. Further basic research into the mechanisms driving research observations of epigenetic-brain correspondence is imperative before any regular clinical application is possible or warranted.

To conclude, this study connects epigenetic ageing with brain structural integrity, cognition, and social determinants of health in virally suppressed PWH and PWoH, and shows that environmental, medical, and social conditions represent modifiable risk factors to promote resilient ageing in this population, highlighting the potential of epigenetic clocks as biomarkers for future clinical care and drug trials.

## Contributors

Conceptualisation: KJP, BMA, THB, AS.

Data curation: SC, MB, AA, BN, PR.

Software: JR, PC.

Formal analysis: KJP, SK.

Funding acquisition: KJP, BMA, EW.

Project administration: EW.

Visualisation: KJP, SK.

Writing–Original Draft: KJP.

Writing–Review and Editing: KJP, BMA, THB, MC, AS.

All authors have reviewed and approved the final manuscript. Multiple authors (KJP, BMA, SC) accessed and verified the underlying data.

## Data sharing statement

De-identified demographic and neurocognitive data from the parent study are available from the NIMH Data Archive (accession no. C5319; https://doi.org/10.15154/6f25-6s63). The authors will share other de-identified participant data including epigenetics and neuroimaging upon reasonable written request including a description of the proposed aims and hypotheses. Shared data will include brain-predicted age and brain age gap (BAG) along with demographics including age, sex, serostatus, race, education level and cognitive testing results (global and domain-specific neuropsychological z-scores). Analysis code used to generate these results is publicly available (https://github.com/KalenJP/HIVClocksProject).

## Declaration of interests

KJP declares K01 and R01 research awards from National Institute of Mental Health, which supported travel. THB participates on the Scientific Advisory Board of Excision BioTherapeutics, and has equity in Excision BioTherapeutics. AS declares grants from the NIH (U24 CA258483, co-I; RF1 MH123163, subcontract PI; R01 MH128286-01A1, co-I; 5R01-MH129832-02, subcontract PI; R01AG067103, PI), intramural funding from the McDonnell Centre for Systems Neuroscience (Small Grants Funding, MPI; Big Ideas Award, MPI), and the BrightFocus Foundation (project A2021042S, PI). AS received payment for participating as a NIH/SREA reviewer for a CSR study section meeting Payment for participating as member of the Alzheimer’s Disease Research Committee Members and reviewing grant applications for BrightFocus Foundation. AS received support for attending meetings (e.g., MICCAI and RSNA conferences during 2023), provided through their Faculty Book & Travel account, and to attend grant review meetings for BrightFocus Foundation. AS holds an issued patent for a method and device for efficient parallel message computation for map inference, and a submitted patent for systems and methods for multiple instance learning for whole slide classification. AS also holds equity in TheraPanacea (23 shares), obtained through participation in a friends-and-family funding round. The other authors declare no competing interests or financial or personal relationships relevant to this work.
