# Supplementary material for: The GrimAge clock corresponds to brain ageing and social determinants of health in people with HIV
Source: eBioMedicine. 2026 Sep 24;132:106492. doi: 10.1016/j.ebiom.2026.106492 (PMC13625693; doi:10.1016/j.ebiom.2026.106492)
Supplement: Supplementary file 1 [file mmc1.docx]

**Fig. S1.** *(Top row)* Regional brain volumes show distinct patterns of association with GrimAge in PWH, with more frontal lobe reductions compared to *(Bottom row)* a more posterior-predominant pattern of GrimAge associations in PWoH. In both groups, GrimAge-brain volume correlations were consistently negative.

**Fig. S2.** Cortical thickness associations with epigenetic aging diverged by HIV serostatus. *(To row)* For PWH, greater GrimAge was not associated with consistently reduced cortical thickness, and slight positive associations were measured. *(Bottom row)* In contrast, associations were larger and more negative for PWoH. This suggests that the predominant neural signature of increased epigenetic age in PWH may be regional volumetric changes rather than cortical gray matter thinning.

**Fig. S3.** GrimAge was associated with poorer executive performance and language function across all participants and in PWoH. However, these associations were not significant after false discovery rate correction. For executive function, effect sizes were similar for PWH and PWoH at -0.3 cognitive *z*-units per decade increased GrimAge.
